# Insights from a high-fat diet fed mouse model with a humanized liver

**DOI:** 10.1371/journal.pone.0268260

**Published:** 2022-05-09

**Authors:** Romil Saxena, Mehdi Nassiri, Xiao-Ming Yin, Núria Morral

**Affiliations:** 1 Department of Pathology and Laboratory Medicine, Indiana University School of Medicine, Indianapolis, Indiana, United States of America; 2 Department of Medical and Molecular Genetics, Indiana University School of Medicine, Indianapolis, Indiana, United States of America; 3 Department of Biochemistry and Molecular Biology, Indiana University School of Medicine, Indianapolis, Indiana, United States of America; University of Illinois, UNITED STATES

## Abstract

Non-alcoholic fatty liver disease (NAFLD) is the most prevalent chronic liver disorder worldwide and is increasing at an alarming rate. NAFLD is strongly associated with obesity and insulin resistance. The use of animal models remains a vital aspect for investigating the molecular mechanisms contributing to metabolic dysregulation and facilitating novel drug target identification. However, some differences exist between mouse and human hepatocyte physiology. Recently, chimeric mice with human liver have been generated, representing a step forward in the development of animal models relevant to human disease. Here we explored the feasibility of using one of these models (cDNA-uPA/SCID) to recapitulate obesity, insulin resistance and NAFLD upon feeding a Western-style diet. Furthermore, given the importance of a proper control diet, we first evaluated whether there are differences between feeding a purified ingredient control diet that matches the composition of the high-fat diet and feeding a grain-based chow diet. We show that mice fed chow have a higher food intake and fed glucose levels than mice that received a low-fat purified ingredient diet, suggesting that the last one represents a better control diet. Upon feeding a high-fat or matched ingredient control diet for 12 weeks, cDNA-uPA/SCID chimeric mice developed extensive macrovesicular steatosis, a feature previously associated with reduced growth hormone action. However, mice were resistant to diet-induced obesity and remained glucose tolerant. Genetic background is fundamental for the development of obesity and insulin resistance. Our data suggests that using a background that favors the development of these traits, such as C57BL/6, may be necessary to establish a humanized mouse model of NAFLD exhibiting the metabolic dysfunction associated with obesity.

## Introduction

The prevalence of non-alcoholic fatty liver disease (NAFLD) is rising at an alarming rate, currently affecting 1 in 4 individuals of the global population [1]. In the early stages, NAFLD is characterized by presence of steatosis without evidence of hepatocellular injury (termed NAFL). Approximately 20% of NAFL patients progress to NASH (steatosis, inflammation with hepatocyte injury, and fibrosis), which can further progress to cirrhosis and liver failure [2, 3]. NAFLD is strongly associated with obesity, metabolic syndrome, and type 2 diabetes [2, 4], all of which are increasing as the result of sedentary lifestyles and consumption of Western pattern diets. In fact, the new term metabolic dysfunction-associated fatty liver disease (MAFLD) has been coined to replace NAFLD, and defines a condition comprising hepatic steatosis and at least one of these three criteria: presence of overweight/obesity, presence of type 2 diabetes, or evidence of metabolic dysfunction [5]. Body fat increases with age and obesity, and is preferentially accumulated in the abdominal region (i.e., visceral adiposity) [6–8]. Abdominal adiposity leads to infiltration of fatty acids into the liver, where production of lipids such as diacylglycerol, impair insulin signaling [9, 10]. Insulin action is critical for the appropriate regulation of gene programs and liver function. Reduced insulin sensitivity in this tissue leads to lack of inhibition of gluconeogenesis and glycogen breakdown, increasing hepatic glucose output [10, 11]. In addition, the *de novo* lipogenesis pathway remains active, exacerbating lipid accumulation, and increasing very-low density lipoprotein (VLDL) secretion and plasma triglycerides. Reduced inhibition of hepatic glucose output and increased lipogenesis/VLDL secretion lead to a combination of hyperglycemia and hypertriglyceridemia, which constitute major risk factors for cardiovascular disease [2, 8, 12, 13]. Currently, there are no FDA-approved therapies to treat NAFLD.

The use of animal models is vital to investigate the molecular mechanisms leading to metabolic dysfunction and facilitate the development of new drug treatments. One of the most utilized models is the C57BL/6 mouse fed a Western-style diet high in fatty acids, cholesterol and sucrose/fructose. This model displays multiple aspects of the metabolic dysfunction seen in human NAFL, including simple steatosis, hyperlipidemia, hyperglycemia, and hyperinsulinemia [14–16], and has provided valuable information on molecular mechanisms contributing to insulin resistance [16–18]. However, some differences exist between human and mouse hepatocyte physiology. For instance, rodents have high levels of high-density lipoproteins (HDL) and low levels of low-density lipoproteins (LDL), while in humans, LDL represents a larger fraction than HDL [19]. Recently, several chimeric mouse models with a liver replaced with transplanted human hepatocytes have been developed [20]. The cDNA-uPA/SCID mouse has a liver repopulated with approximately 80–85% of human hepatocytes [21]. Mice are generated by transplanting human hepatocytes via the spleen into severe combined immunodeficient (SCID) mice expressing urokinase-type plasminogen activator (uPA) driven by the albumin enhancer/promoter. Lipoprotein profiles are similar to those in humans, and hepatocytes express mRNAs for a variety of human cytochrome P450 (hCYP) subtypes [19, 22, 23]. The human hepatocytes of chimeric mice are in a growth hormone (GH) deficient state due to low binding of mouse GH to the human receptor [21, 24], which results in activation of the lipogenesis program. Thus, this mouse model could be useful to study the pathophysiological mechanisms associated with lipid accumulation in human hepatocytes. However, it is unknown whether these mice can develop metabolic dysfunction and features characteristic of human NAFLD which are present in the C57BL/6 mouse model fed a Western-style diet, including obesity, insulin resistance, and glucose intolerance.

Here we have explored the feasibility of inducing obesity and its associated metabolic dysfunction by feeding cDNA-uPA/SCID mice with a high-fat diet. Previous studies have pointed at the importance of choosing the appropriate control diet in metabolic disease research [25]. Thus, we have first assessed whether differences exist between feeding a purified ingredient diet that matches the composition of the high-fat diet, and feeding a chow diet, as there are marked differences in micro- and macronutrient content between the two [26].

## Materials and methods

### Animals and diets

All animal procedures were conducted in accordance with the National Institutes of Health guidelines, and approval was obtained from the Institutional Animal Care and Use Committee of Indiana University School Medicine (#11027 and #18073). Five-week old male C57BL6/J mice were purchased from The Jackson Laboratory (Bar Harbor, ME). Male cDNA-uPA/SCID mice (also known as PXB) [27] were purchased from PhoenixBio Co., Ltd. (New York, NY). The development and characterization of this mouse model has been extensively described [21, 27]. Briefly, at 3 weeks of age cDNA-uPA/SCID mice received cryopreserved hepatocytes from a 1-year-old Caucasian male human donor obtained from a commercial vendor (Lot: JFC; BioIVT, Westbury, NY) by injection into the spleen [27]. At 16 weeks, mice had an estimated replacement index >80%, based on human serum albumin levels [27]. Mice were 18 weeks old when they arrived at our animal facility.

Mice were allowed to acclimate in a BSL1 (C57BL/6) or a BSL2 room (cDNA-uPA/SCID mice) for 10–14 days. A standard 12 h light/12 h dark cycle (7 AM/7 PM) was maintained throughout the experiments. C57BL/6 mice were 7 weeks old at the time they started receiving one of the following diets (n = 5): (i) high-fat diet (D12492, Research Diets: 60 kcal% fat, 20% protein, 20% carbohydrate; contains 7% kcal from sucrose, as well as 279.6 mg/kg cholesterol from lard; 5.24 kcal/g); (ii) a matched control diet (D12450K, Research Diets: 10 kcal% fat, 20% protein, 70% carbohydrate; does not contain fructose and has 51.6 mg/kg of cholesterol; 3.85 kcal/g); or (iii) chow diet (2018SX, Envigo: 18% kcal fat, 24% protein, 58% carbohydrate; 3.1 kcal/g). cDNA-uPA/SCID mice were 20 weeks old when they started receiving irradiated diets D12492i or D12450Ki (Research Diets) (n = 4). Mice were fed *ad libitum*, and allowed free access to water. Fresh pellets were provided on a weekly basis, and body weight was monitored at that point. Mice were fed a special diet for 10 weeks (C57BL/6) or 12 weeks (cDNA-uPA/SCID), and were euthanized by decapitation under fed conditions. Blood glucose measurements under fed conditions were taken at 9:30 am– 10:30 am. Tissues were collected and snap frozen in liquid nitrogen, embedded in OCT and frozen, or fixed in 10% buffered formalin for histology analysis.

Average food consumption of C57BL/6 mice was estimated by measuring leftover food from the previous week (8 total measurements), divided by number of mice in the cage. Calorie intake was obtained by multiplying food intake (in g) by the energy density (kcal/g) provided by the supplier.

### Glucose Tolerance (GTT) tests

The GTT was performed by intraperitoneal administration of a bolus of glucose (1 mg/g body weight), after an overnight (C57BL/6) or a 7-hour fast (cDNA-uPA/SCID mice). Blood glucose was monitored from a tail vein drop after 15, 30, 60 and 120 minutes, using a AimStrip Plus glucose meter (Germaine Laboratories, San Antonio, TX), as described [28, 29].

### Tissue lipids

Liver lipid analysis was carried out using ~100 mg of frozen liver tissue. Total liver triglyceride, free fatty acids, and cholesterol, were analyzed by thin layer chromatography and gas chromatography at the Mouse Metabolic Phenotyping Center at Vanderbilt University, as previously described [29].

### Tissue histology

For histology, tissues were processed as previously described [28]. Liver, spleen and kidney were fixed in 10% buffered formalin, and processed in paraffin. Four-micron thick sections were cut from routinely processed paraffin embedded tissue and stained with hematoxylin and eosin for histological examination. Sections of liver tissue snap-frozen in OCT compound were stained with Oil Red O (ORO), and the percentage of hepatocytes containing lipid droplets was estimated. Macrovesicular steatosis was defined as the presence of a single intracellular droplet of fat, irrespective of the size of the lipid droplet. Processing and staining were performed by the Immunohistochemistry Core (IHC) at Indiana University School of Medicine.

### Serum biochemistries

All serum biochemistries were analyzed by the Center for Diabetes and Metabolic Diseases Translational Core. Insulin was analyzed using a mouse insulin ELISA kit (#10-1247-10, Mercodia, Uppsala, Sweden); alanine aminotransferase (ALT) was analyzed with a kit from Randox (#AL 3875), which uses a chemistry that allows quantifying ALT irrespective of species (mouse or human). All sample measurements were carried out in duplicate. Triglycerides, cholesterol and free fatty acids (FFA) were measured using a Randox Daytona Clinical Chemistry Analyzer (#TR3823, #CH3810, and #FA115, respectively).

### Statistical analysis

Data are presented as the arithmetic mean ± standard deviation. *P* values were calculated using unpaired two-tailed Student’s *t*-tests using Microsoft Excel v16.54. A *P* value of less than 0.05 was considered statistically significant.

## Results

### Chow versus refined control diet

**Fig 1:**
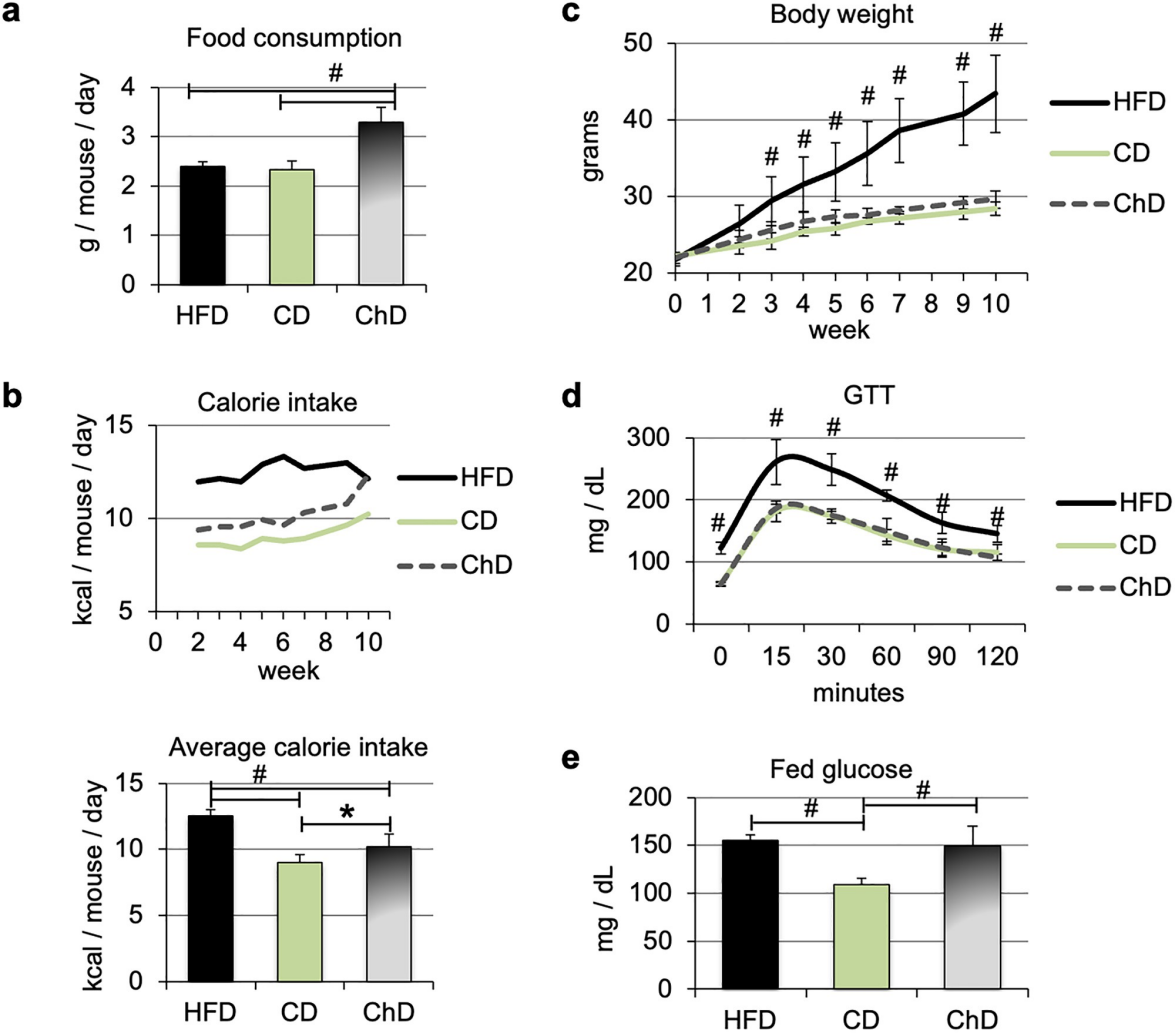
Increased body weight gain and development of glucose intolerance in high fat diet fed C57BL/6 mice. C57BL/6 mice were fed a HFD, a matched purified ingredient control diet (CD), or a regular chow diet (ChD) for 10 weeks. **(a)** Average food consumption; ^#^p<0.01 (n = 5); **(b)** Calorie intake throughout the experimental period and average intake *p<0.05, ^#^p<0.01 (n = 5); **(c)** Body weight gain. Starting from week 3, body weights between HFD-fed and CD were significantly different at p<0.01; HFD versus ChD groups were also significantly different, although weeks 3, 4 and 5 at a lower p value (p<0.05); **(d)** Glucose tolerance test. Mice were fasted overnight and given a bolus of glucose (1 mg/g body weight). Blood glucose was monitored at the indicated time points. The indicated time points in the HFD-fed group were significantly different relative to the CD as well as ChD groups; **(e)** Fed blood glucose after 10 weeks of feeding the special diets; ^#^p<0.01 (n = 5).

In C57BL/6 mice, high-fat feeding using purified ingredient diets induces insulin resistance and glucose intolerance within 8 weeks [14–16, 30]. Nutrients from plant sources present in chow diets and absent in purified ingredient diets influence multiple traits, including food intake, hormone levels (insulin, leptin), and body and adipose tissue weight [31, 32]. To address potential differences in food consumption and glucose tolerance between control diets, groups of C57BL/6 mice were fed a diet containing 60% kcal from fat (HFD), a matched control diet with 10% kcal from fat (CD), or a chow diet (18% kcal from fat, ChD) for 10 weeks. Remarkably, mice that were fed the chow diet ate more and had a slightly higher calorie intake (average 10.2 kcal/day/mouse) than mice fed a matched purified ingredient control diet (average 9.01 kcal/day/mouse) (Fig 1a and 1b). No difference in body weight was observed between the ChD and CD diets, despite the higher energy intake in the chow group (Fig 1c). In contrast, mice that received the HFD had similar food consumption (in grams) to mice fed the matched ingredient CD, and an average energy intake of 12.51 kcal/day/mouse (Fig 1a and 1b). As anticipated, mice in the HFD group gained significantly more weight than mice in the control diets, starting at 3 weeks of HFD feeding (Fig 1c).

Glucose tolerance tests were carried out on week 9 to confirm the presence of glucose intolerance in the HFD group (Fig 1d). As anticipated, these mice had significantly higher blood glucose levels at all time points, indicating they were glucose intolerant. No difference in glucose tolerance was observed between the ChD and CD control diets. Furthermore, fasting glucose of both control groups was significantly lower than in the HFD group (Fig 1d, time 0). At the end of the study, blood glucose levels under fed conditions were monitored. Mice that received the HFD had significantly higher blood glucose levels than the CD group (Fig 1e). However, fed blood glucose in the chow group was similar to the HFD, suggesting that the chow and purified ingredient control diets elicit distinct responses. Serum levels of insulin, triglycerides, fatty acids, cholesterol, and ALT were not different between the control diets (Table 1). Insulin and cholesterol levels were significantly higher in the HFD relative to the two control diets, as anticipated. ALT was significantly higher when compared to the matched control diet, but not relative to the chow diet (Table 1).

**Table 1:** Non-fasting serum chemistries.

	Insulin (ng/ml)	FFA (mmol/l)	Cholesterol (mmol/l)	TG (mmol/l)	ALT (U/l)
**C57BL/6**					
HFD	4.68 ± 1.77*^,^**	ND	3.57 ± 0.14*^,^**	0.98 ± 0.08	50 ± 19.04*
CD	1.84 ± 1.13	ND	2.26 ± 0.24	1.15 ± 0.31	27 ± 9.75
ChD	1.53 ± 0.80	ND	2.03 ± 0.23	1.07 ± 0.23	30 ± 7.07
**cDNA-uPA/SCID**					
HFD	0.52 ± 0.59	0.66 ± 0.16	1.11 ± 0.20	0.67 ± 0.16	63.75 ± 6.08
CD	0.29 ± 0.13	0.63 ± 0.27	1.12 ± 0.41	0.57 ± 0.20	89.5 ± 59.16

n = 5, C57BL/6; n = 4, cDNA-uPA/SCID mice;

*p<0.05 relative to CD;

**p<0.05 relative to chow;

ND: not determined

**Fig 2:**
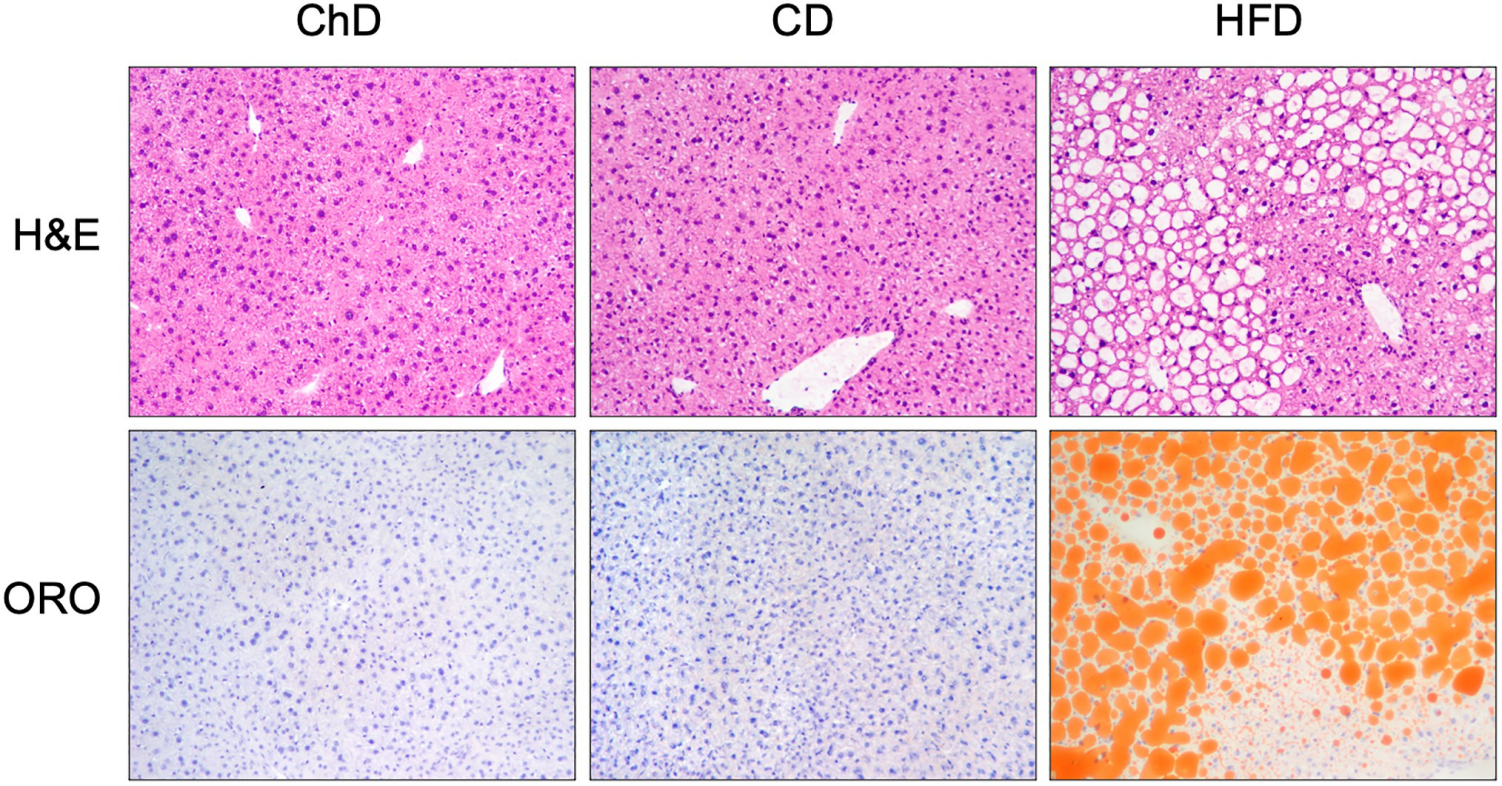
Hepatic steatosis in C57BL/6 mice fed a HFD. Livers from mice fed the CD or ChD diet showed no lipid accumulation. Mice fed the HFD had marked extensive macrovesicular steatosis. Hematoxylin & eosin, 100x; Oil Red O, 100x.

Liver sections stained with hematoxylin-eosin and Oil-Red-O showed no lipid accumulation in mice receiving the CD or ChD diets, and had undistinguishable pathology profiles. In contrast, mice that received the HFD demonstrated macrovesicular steatosis in 80–90% of hepatocytes, as anticipated (Fig 2).

Overall, our data showed that the chow diet and the purified ingredient control diet supported body weight to a similar extent in mice, despite a slightly higher calorie intake in chow-fed mice. Glucose levels under fed conditions were higher in the last group. The fact that we observed differences between the two control diet groups indicates that the purified ingredient diet is more appropriate as control diet than chow.

### High-fat feeding in mice with a humanized chimeric liver

**Fig 3:**
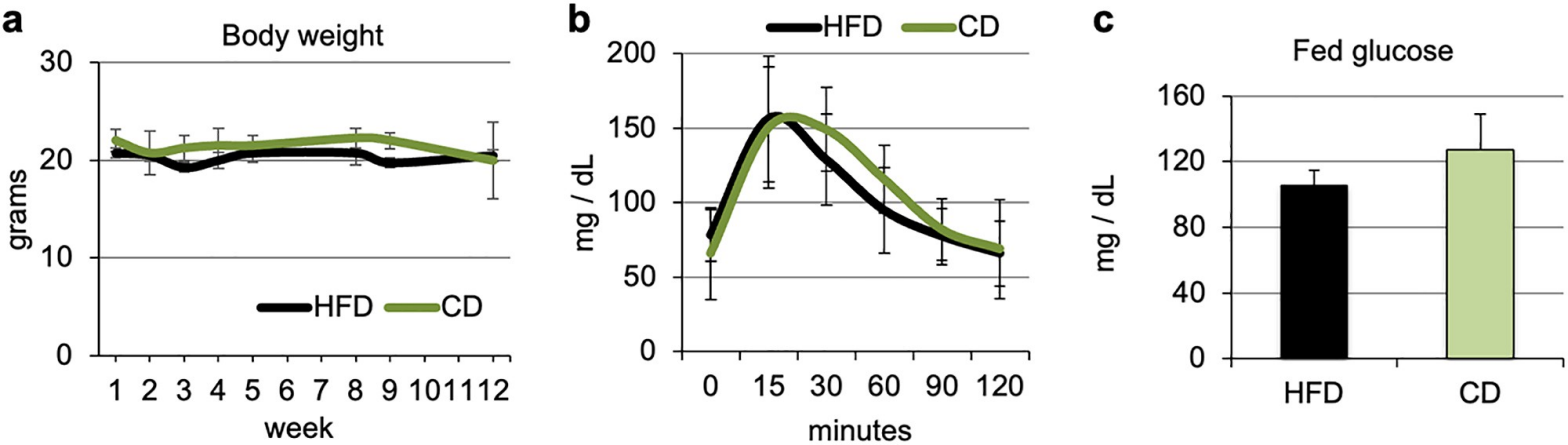
cDNA-uPA/SCID mice do not develop insulin resistance upon HFD feeding. cDNA-uPA/SCID mice were fed a HFD or CD for 12 weeks. **(a)** Body weight (n = 4); **(b)** GTT on week 12. Mice were fasted for 7 hours and a bolus of glucose (1 mg/g body weight) was given intraperitoneally. Blood glucose was measured at the indicated times (n = 4); **(c)** Fed blood glucose was measured 2 days after the GTT (n = 4).

We then questioned whether cDNA-uPA/SCID mice fed a HFD would develop obesity and the associated metabolic dysfunction, including hyperinsulinemia and glucose intolerance. Mice were fed the HFD or CD for 12 weeks. Unlike C57BL/6 mice, cDNA-uPA/SCID mice did not develop obesity, and body weight remained similar in both groups for the entire period (Fig 3a). Glucose tolerance tests were performed at week 12. Consistent with the lack of obesity, cDNA-uPA/SCID mice fed a HFD did not develop glucose intolerance, and responded similarly to a glucose challenge than mice fed a CD (Fig 3b). Additionally, blood glucose in the fed state was not significantly differently between the diets (Fig 3c). Serum insulin, free fatty acids (FFA), cholesterol, triglycerides, and ALT, were not significantly different between CD and HFD fed mice (Table 1).

**Fig 4:**
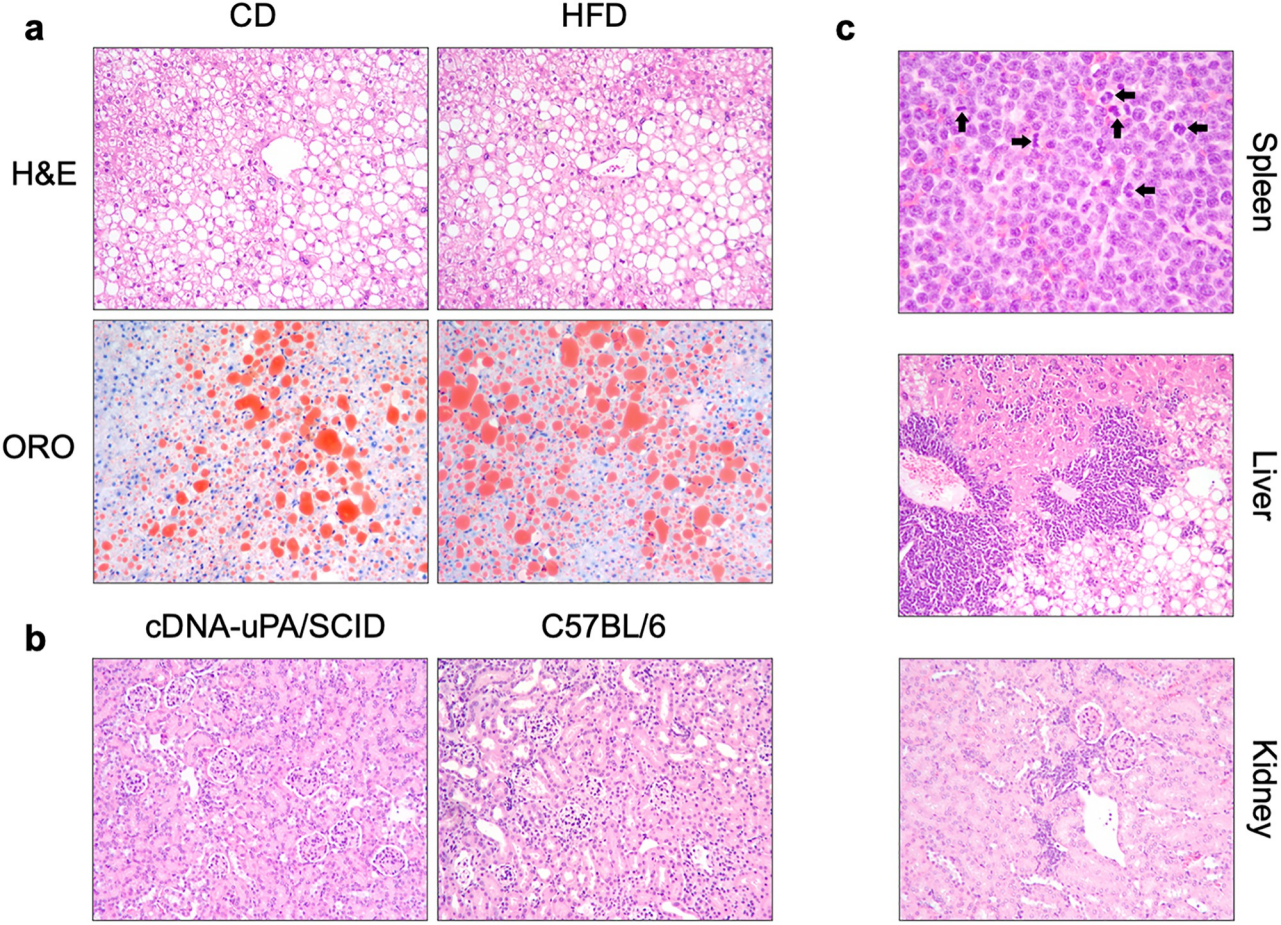
Liver and kidney histology in cDNA-uPA/SCID mice. **(a)** cDNA-uPA/SCID mice fed a HFD or a CD develop hepatic steatosis regardless of diet. Similar amounts of lipid droplets were observed in the ORO staining. **(b)** Kidney abnormalities in cDNA-uPA/SCID mice. Glomeruli in kidneys of cDNA-uPA/SCID mice showed an increase in the mesangial matrix with narrowing of the capillary loops, and variable increase in size, as compared to C57BL/6 mice. Hematoxylin eosin, 100x; Oil Red O, 100x. **(c)** Lymphoma in cDNA-uPA/SCID mice. The normal splenic architecture is effaced by an infiltrate of medium sized, hyperchromatic, monotonous cells with a high nuclear-cytoplasmic ratio and scant cytoplasm. The nuclei displayed irregular and coarse membranes, and contained clumped chromation with inconspicious nucleoli. Numerous mitotic figures (arrows) are present (top). The liver (middle) is massively involved and the kidney (bottom) shows a similar infiltrate of monotonous, dyscohesive, hyperchromatic cells.

The lack of growth hormone on the human hepatocytes of cDNA-uPA/SCID chimeric mice has been shown to activate the lipogenesis program [21, 24]. To assess the effects after extended periods of time (>200 days; mouse age 3 to 32 weeks old), liver sections were stained with hematoxylin/eosin and Oil Red O. Most hepatocytes displayed fat vacuoles in the livers of mice treated with HFD and CD, with only 10–15% of hepatocytes without accumulation of fat. Macrovesicular steatosis was present in 80–90% of hepatocytes, and lipid droplet size was similar between the two groups (Fig 4a). Liver lipid analysis showed no differences in FFA, triglyceride, and cholesterol content (Table 2). Nonetheless, significant changes in fatty acid composition were observed. Relative to the CD, triglycerides in HFD-fed mice had more representation of 16:1 and 18:1w7, and lower levels of 18:2 (Table 2). Conversely, in the pool of FFA, 16:1 and 18:1w7 were less represented, while 18:2 was more abundant. Thus, the HFD shifted the fatty acid composition in triglycerides and FFA in the liver.

**Table 2:** Liver lipids in cDNA-uPA/SCID mice.

	FFA		TG		Cholesterol	
	HFD	CD	HFD	CD	HFD	CD
**μg/mg**:	1.4 ± 0.2	1.2 ± 0.2	72.9 ± 21.0	74.7 ± 23.5	2.2 ± 0.3	3.1 ± 1.8
14:0	0.7 ± 0.2	1.0 ± 0.3	1.1 ± 0.2	0.7 ± 0.2		
16:0	28.7 ± 0.9	29.3 ± 2.8	22.3 ± 2.8	21.7 ± 2.0		
16:1	0.9 ± 0.1*	3.5 ± 0.5	4.2 ± 1.7*	1.2 ± 0.0		
18:0	18.4 ± 1.8*	15.5 ± 1.1	5.8 ± 1.0	6.3 ± 0.5		
18:1w9	23.0 ± 1.3	24.0 ± 2.6	43.6 ± 3.0	43.3 ± 1.0		
18:1w7	1.7 ± 0.1*	3.5 ± 0.7	4.5 ± 0.4*	2.2 ± 0.2		
18:2	16.1 ± 0.6*	13.6 ± 1.7	16.1 ± 3.3*	22.2 ± 0.7		
18:3w3	-	-	0.8 ± 0.1	0.9 ± 0.1		
20:3w6	-	-	0.4 ± 0.3	0.4 ± 0.2		
20:4	7.1 ± 0.8	6.1 ± 1.3	0.7 ± 0.5	0.8 ± 0.1		
22:6	3.2 ± 0.1	3.4 ± 0.4	0.5 ± 0.5	0.4 ± 0.2		

*p<0.05 relative to CD; n = 4

### Kidney and spleen pathology in cDNA-uPA/SCID mice

Previous studies have shown that chimeric uPA/SCID and non-transplanted uPA/SCID mice develop kidney disorders, which is believed to be due to high uPA activity in fetal or neonatal mice blood [27]. This abnormality was improved or eliminated in cDNA-uPA/SCID mice [27]. Our data indicates that cDNA-uPA/SCID mice can develop kidney abnormalities to some degree. Glomeruli in kidneys of cDNA-uPA/SCID mice showed an increase in the mesangial matrix with narrowing of the capillary loops, accompanied variably by an increase in glomerular size (Fig 4b).

Lastly, we observed that the spleens of cDNA-uPA/SCID mice were involved by lymphoma. The lymphoma was characterized as a proliferation of hyperchromatic, large monotonous cells with a high nuclear-cytoplasmic ratio and scant cytoplasm. The nuclei displayed irregular and coarse membranes, and contained clumped chromation with inconspicious nucleoli (Fig 4c). Numerous mitotic figures (arrows) were present. The livers and kidneys in 2 mice (one in the CD group and the second in the HFD group) were involved by a similar infiltrate of monotonous, dyscohesive, hyperchromatic cells (Fig 4c).

## Discussion

Metabolic dysfunction-associated fatty liver disease is one of the major health challenges of the 21st century, and is rising to an alarming proportion. Aging of the global population together with high obesity rates, promoted by sedentarism and overnutrition, are major contributors to its growing prevalence. Obesity is a major contributor to NAFLD and is strongly linked to insulin resistance. Mouse models that recapitulate the metabolic abnormalities seen in humans are vital to study the molecular mechanisms that contribute to the underlying pathophysiology. Current experimental murine models of diet-induced NAFLD are not the most accurate predictors of human liver metabolism because differences exist between human and mouse hepatocyte physiology [33]. Thus, humanized animal models represent instrumental tools to carry out preclinical studies.

The Western-style diet fed mouse is the most utilized model of diet-induced obesity with features of metabolic dysfunction [15, 16]. To be able to make conclusions on the effects of macronutrients, an appropriate control diet should be used. Chow diets are composed of natural ingredients, including ground wheat and ground corn, soybean meal, corn gluten meal, and wheat middlings, and contain phytoestrogens, which confer protective effects on lipid homeostasis, and improve glucose control and insulin sensitivity [34–36]. Indeed, feeding rats a diet containing 600 μg/g of phytoestrogens increased food consumption, and at the same time, lowered insulin and body weight relative to rats fed a diet low in phytoestrogens [32]. Rats also had higher circulating T3 thyroid hormone levels and a tendency for increased non-fasting glucose levels [32]. Consistent with these data in rats, our data in mice showed that food intake was higher in animals fed a chow diet relative to the purified ingredient control diet. Nevertheless, the two groups of mice displayed comparable weight gain. Also, higher non-fasting glucose levels were observed in chow fed mice, despite the fact that insulin was not different between the two groups. Fasting glucose levels were undistinguishable between the two control diets, an indication of comparable hepatic glucose output. Neither control diet contained sucrose, a simple sugar that has been linked to lipogenesis, dyslipidemia and insulin resistance [37, 38]. In contrast, the HFD contained 7% of kcal as sucrose, in addition to high-fat (60% of kcal) and cholesterol (from lard), which together contributed to metabolic dysfunction in C57BL/6 mice, including steatosis, hyperinsulinemia, glucose intolerance, increased fasting and fed blood glucose, and high serum cholesterol.

In sharp contrast to the C57BL/6 mouse, feeding cDNA-uPA/SCID mice with a HFD did not increase body weight, and did not elicit hyperinsulinemia, glucose intolerance, or changes in fasting blood glucose. Thus, this mouse is resistant to the development of obesity and insulin resistance. It is well known that genetic differences between mouse strains play an important role in the development of metabolic dysfunction [14, 39]. Previous studies have shown that some strains are susceptible to developing obesity, hyperglycemia and hyperinsulinemia, while others are not [14, 40]. The genetic background of the cDNA-uPA/SCID mouse is 70–75% C.B-17 and 25–30% SJL, B6, and 129SvEv [27]. It is possible that this mixed strain confers resistance to the development of obesity. Actually, previous studies in immunodeficient mice attempting to develop the metabolic syndrome by feeding a HFD have underlined the importance of mouse background. Severe combined immunodeficiency (SCID), non-obese diabetic/severe combined immunodeficiency (NOD/SCID) and NOD/SCID-IL2Rγ (NSG) mice are resistant to developing metabolic syndrome [41–43]. Nevertheless, mice with inactivating mutations in the recombination activating gene 1 (*Rag1*) backcrossed onto the C57BL/6 background, lack mature B and T lymphocytes, and yet, develop obesity, insulin resistance and hyperglycemia upon feeding a high-fat diet [44–46]. Indeed, *Rag1*-/- mice fed a high-fat diet (42.2% kcal as fat) for 11 weeks gained more weight than C57BL/6 mice fed the same diet, and developed similar hyperinsulinemia and impaired glucose tolerance [44]. Furthermore, *Rag1*-/- mice developed insulin resistance, with impaired glucose and insulin tolerance, hyperinsulinemia and hyperglycemia within a week of feeding a diet with 60% kcal as fat [45, 47]. In long-term studies, feeding *Rag1*-/- mice a diet containing 45% kcal as fat for 28 weeks led to severe metabolic dysfunction relative to a control diet, including larger adipocyte size, intramyocellular lipid content, hepatic steatosis, insulin resistance, hyperinsulinemia, and impaired glucose tolerance [46]. Thus, the C57BL/6 genetic background favors the development of obesity and the associated metabolic dysfunction. A recent study has shown that *Il2rg*^-/-^/*Rag2*^-/-^/*Fah*^-/-^ mice with a humanized liver fed a high fat diet (41% kcal fat) developed higher steatosis than mice fed a control diet [48]. Concurring with our results, this mouse model did not develop obesity or increased fasting glucose, which was claimed on the genetic background of the mice [48].

Despite the lack of obesity, our data showed the presence of widespread steatosis independent of diet. Feeding a HFD did not accelerate the degree of lipid accumulation relative to the control diet, and only small differences on the composition of liver lipids were observed. These differences mainly consisted of higher monounsaturated fatty acid representation in triglycerides (16:1 and 18:1w7), at the expense of polyunsaturated fatty acid enrichment (18:2). Although the changes are small, they are consistent with previous studies in mice fed a HFD showing enrichment of monounsaturated and lower polyunsaturated fatty acid representation [49]. In mice, liver-specific growth hormone (GH) receptor deficiency leads to alterations in lipid and glucose metabolism [50]. It has been reported that the human hepatocytes of a chimeric mouse are in a GH-deficient state due to low binding of the mouse GH to the human receptor [21, 24]. Similar to mice with GH receptor deficiency, lack of GH action in human hepatocytes leads to activation of the lipogenesis program, with increased levels of the transcription factor Sterol Regulatory Element Binding Protein 1 and a few of its gene targets. Lipid droplets appear in the cytoplasm of human hepatocytes starting approximately 70 days after transplantation [24]. Mice in our experiments were transplanted 7 months prior to analysis, and thus, human hepatocytes were exposed to GH deficiency for >200 days, exacerbating lipogenesis. Patients with NAFLD have reduced hepatic growth hormone action [51–53]. Thus, the cDNA-uPA/SCID mouse represents a unique *in vivo* experimental tool to study the effects of reduced GH action on human hepatocytes and decipher the molecular aspects underlying the pathophysiology.

The development of kidney and spleen abnormalities seen in cDNA-uPA/SCID mice is a limitation for future development of a humanized mouse model of obesity and insulin resistance, a lengthy process that entails first, engraftment of human hepatocytes for approximately 3 months, and then, feeding special diets for 10–12 weeks to promote the development of metabolic disease, bringing mouse age to >6 months at the end of the experimentation. Despite previous work showing that cDNA-uPA/SCID mice do not develop kidney disease [27], our study shows that 8-month old cDNA-uPA/SCID mice can develop kidney abnormalities, suggesting mouse age increases the risk. Furthermore, we observed that all mice had spleens with lymphoma, and in two cases, the lymphoma had extended to liver and kidney. SCID mice have a mutation in the gene encoding the catalytic subunit of DNA-dependent protein kinase (*Prkdc*), a protein that plays a role in the repair of double-stranded DNA breaks, which makes them susceptible to spontaneous development of malignancies [54] and to develop thymic lymphomas with aging [55]. In this regard, it is worth noting that the presence of these abnormalities has not been described in *Rag1*/*2*-deficient mice [56], and therefore, these immunodeficient mice might be better for generating a model of diet-induced obesity and insulin resistance with a humanized liver.

In summary, our data indicate that differences exist in food consumption and blood glucose between mice fed chow or a diet with matched ingredients to those in the high fat diet. Thus, the last one is a more appropriate control diet in diet-induced obesity studies. cDNA-uPA/SCID mice are resistant to developing obesity, and feeding a high fat diet does not recapitulate the hyperglycemia, hyperinsulinemia and glucose intolerance that is seen in C57BL/6 mice. Nevertheless, cDNA-uPA/SCID mice display widespread steatosis, and may be useful for studies focusing on the effects of growth hormone deficiency and the molecular mechanisms leading to lipid accumulation in human hepatocytes. Given that cDNA-uPA/SCID mice developed additional pathological features in kidney and spleen, using other immunodeficient mice in the C57BL/6 background might be a better approach to develop a humanized model of obesity, insulin resistance and NAFL to enable studies on the effects of hyperinsulinemia, hyperlipidemia and glucotoxicity on human hepatocytes.

## Supporting information

S1 Raw dataset Table 1. Non-fasting serum chemistries.
(PDF)

S2 Raw dataset Table 2. Liver lipids in cDNA-uPA/SCID mice.
(PDF)

S3 Raw dataset Fig 1. C57BL/6 mice fed a HFD or control diets. **(a)** Average food consumption **(b)** Calorie intake throughout the experimental period and average intake; **(c)** Body weight gain; **(d)** Glucose tolerance test; **(e)** Fed blood glucose after 10 weeks of feeding the special diets.
(PDF)

S4 Raw dataset Fig 3. cDNA-uPA/SCID mice fed a HFD or control diet. **(a)** Body weight; **(b)** GTT on week 12; **(c)** Fed blood glucose.
(PDF)
