# Supplementary material for: Insights from a high-fat diet fed mouse model with a humanized liver
Source: PLoS One. 2022 May 9;17(5):e0268260. doi: 10.1371/journal.pone.0268260 (PMC9084523; doi:10.1371/journal.pone.0268260)
Supplement: S1 Raw dataset — Non-fasting serum chemistries. (PDF) [file pone.0268260.s001.pdf]

TABLE 1-C57BL6

	Insulin ng/ml	Chol mmol/l	TG mmol/l	ALT U/l
HFD	6.997	3.35	1	40
	2.618	3.55	1.05	40
	5.617	3.75	0.9	65
	3.274	3.6	1.05	30
	4.915	3.6	0.9	75
CD	1.405	2.2	0.9	30
	0.924	2.1	0.75	40
	1.387	2.6	1.3	15
	3.804	2	1.5	30
	1.704	2.4	1.3	20
Chow	1.419	2	1	30
	2.935	2.4	1.4	30
	1	2	0.95	30
	1.13	1.75	0.8	20
	1.144	2	1.2	40

TABLE 1-PXB

	Insulin ng/ml	FFA mmol/l	Cholesterol mmol/l	TG mmol/l	ALT U/l
HFD	0.079	0.48	1.4	0.9	69
	0.205	0.56	1	0.51	65
	0.428	0.76	1.01	0.64	66
	1.387	0.82	1.01	0.64	55
CD	0.344	0.61	0.87	0.58	62
	0.248	0.86	1	0.62	64
	0.429	0.26	1.72	0.31	178
	0.136	0.8	0.88	0.78	54
