# Supplementary material for: Insights from a high-fat diet fed mouse model with a humanized liver
Source: PLoS One. 2022 May 9;17(5):e0268260. doi: 10.1371/journal.pone.0268260 (PMC9084523; doi:10.1371/journal.pone.0268260)
Supplement: S2 Raw dataset — Liver lipids in cDNA-uPA/SCID mice. (PDF) [file pone.0268260.s002.pdf]

TABLE 2-LIVER LIPIDS

	CD				HFD			
**FFA**								
**Liver**								
**14:0**	1.15	1.25	0.99	0.60	0.81	0.81	0.61	0.51
**16:0**	30.44	28.55	32.28	25.77	28.73	29.89	27.85	28.20
**16:1**	4.02	3.62	3.61	2.78	1.01	0.95	0.95	0.86
**18:0**	15.28	14.89	14.70	17.14	18.46	17.15	17.11	21.04
**18:1w9**	23.42	27.81	22.43	22.32	22.97	22.04	24.91	22.26
**18:1w7**	3.12	4.52	3.05	3.39	1.75	1.73	1.70	1.58
**18:2**	13.62	11.49	13.55	15.69	16.04	16.09	16.77	15.30
**20:4**	5.38	5.06	5.89	8.04	7.10	8.04	6.19	7.21
**22:6**	3.58	2.82	3.50	3.56	3.13	3.29	3.26	3.04
**Total ug**	116.5	109.7	136.7	139.1	119.1	134.1	157.0	151.9
**mg tissue**	113.0	98.0	97.0	110.0	105.0	104.0	104.0	99.0
**ug/mg**	1.03	1.12	1.41	1.26	1.13	1.29	1.51	1.53
**TG**								
**Liver**								
**14:0**	0.59	0.60	0.78	0.97	1.16	0.77	1.27	1.09
**16:0**	20.60	19.51	23.71	22.85	24.78	19.82	24.61	19.98
**16:1**	1.13	1.18	1.16	1.14	4.42	3.89	6.32	2.30
**18:0**	6.86	6.31	6.48	5.59	4.49	6.21	5.83	6.77
**18:1w9**	43.30	44.71	42.29	42.86	47.37	44.58	41.29	41.17
**18:1w7**	2.08	2.24	2.52	2.06	4.50	4.50	4.02	4.91
**18:2**	22.70	22.78	21.36	22.09	12.28	17.79	14.61	19.69
**18:3 w3**	0.98	0.91	0.77	0.83	0.63	0.74	0.96	0.69
**20:3w6**	0.49	0.50		0.42		0.47	0.40	0.82
**20:4**	0.76	0.79	0.93	0.75	0.38	0.76	0.42	1.44
**22:6**	0.50	0.47		0.44		0.48	0.27	1.13
**Total ug**	11104.4	6769.4	4299.0	9581.9	7894.4	6596.5	10506.3	5146.4
**mg tissue**	113.0	98.0	97.0	110.0	105.0	104.0	104.0	99.0
**ug/mg**	98.27	69.08	44.32	87.11	75.18	63.43	101.02	51.98
**T. Chol**								
**Liver**								
**Total ug**	211.7	320.1	169.0	622.8	241.8	226.3	189.9	261.9
**mg tissue**	113.0	98.0	97.0	110.0	105.0	104.0	104.0	99.0
**ug/mg**	1.87	3.27	1.74	5.66	2.30	2.18	1.83	2.65
