# Supplementary material for: Insights from a high-fat diet fed mouse model with a humanized liver
Source: PLoS One. 2022 May 9;17(5):e0268260. doi: 10.1371/journal.pone.0268260 (PMC9084523; doi:10.1371/journal.pone.0268260)
Supplement: S3 Raw dataset — C57BL/6 mice fed a HFD or control diets. (a) Average food consumption (b) Calorie intake throughout the experimental period and average intake; (c) Body weight gain; (d) Glucose tolerance test; (e) Fed blood glucose after 10 weeks of feeding the special diets. (PDF) [file pone.0268260.s003.pdf]

Fig. 1A, 1B

**WEEK:**	**2**	**3**	**4**	**5**	**6**	**7**	**9**	**10**	
g consumed/mouse/day									
HFD	2.29	2.31	2.29	2.46	2.54	2.42	2.48	2.3	FIG 1A
CD	2.23	2.23	2.17	2.31	2.29	2.31	2.50	2.7	
CHOW	3.03	3.09	3.09	3.20	3.11	3.33	3.48	4.0	
calorie intake/mouse/day									
HFD	12.0	12.1	12.0	12.9	13.3	12.7	13.0	12.1	FIG 1B
CD	8.6	8.6	8.4	8.9	8.8	8.9	9.6	10.2	
CHOW	9.4	9.6	9.6	9.9	9.7	10.3	10.8	12.3	

Fig. 1C

WEEK:	0	2	3	4	5	6	7	9	10
HFD	21	24	26	28	29	31	34	35	36
D12492	21	25	27	28	30	32	35	40	42
	22	25	29	32	33	36	39	40	43
	22	28	31	34	36	38	41	43	47
	23	30	34	36	38	41	44	46	49
CD	22	22	23	25	25	26	26	27	27
D12450K	22	23	23	25	25	27	27	27	28
	22	24	25	25	26	27	27	28	29
	22	24	25	26	26	27	28	29	29
	23	25	25	26	27	27	28	29	29
ChD	21	23	24	25	27	27	28	28	28
chow	22	24	25	27	27	27	28	29	29
	22	24	26	27	27	27	28	29	30
	22	25	26	27	27	28	28	30	30
	23	26	27	28	29	29	29	30	31

Fig. 1D

	0'	15'	30'	60'	90'	120'
HFD	119	302	248	211	155	141
D12492	120	282	261	202	151	131
	138	209	208	204	158	142
	121	243	252	221	193	167
	112	272	276	198	158	146
CD	70	171	182	140	118	113
D12450K	60	197	182	156	136	135
	67	196	179	141	124	116
	62	159	156	145	120	99
	65	184	169	130	102	113
CHOW	66	193	169	161	139	113
	67	189	169	112	105	99
	65	191	175	164	117	110
	61	178	179	152	123	106
	63	180	183	156	132	110

Fig. 1E

	Fed blood glucose (mg/dl)
HFD	156
D12492	152
	151
	165
	153
CD	108
D12450K	113
	100
	108
	118
ChD	141
Chow	140
	131
	153
	183
