# Supplementary material for: Insights from a high-fat diet fed mouse model with a humanized liver
Source: PLoS One. 2022 May 9;17(5):e0268260. doi: 10.1371/journal.pone.0268260 (PMC9084523; doi:10.1371/journal.pone.0268260)
Supplement: S4 Raw dataset — cDNA-uPA/SCID mice fed a HFD or control diet. (a) Body weight; (b) GTT on week 12; (c) Fed blood glucose. (PDF) [file pone.0268260.s004.pdf]

Fig. 3A

week	HFD (g)	CD (g)
1	21	21
	20	21
	21	23
	21	23
2	20	20
	20	18
	21	23
	21	22
3	20	21
	19	20
	19	21
	19	23
4	21	20
	20	20
	19	23
	20	23
5	22	22
	20	20
	20	22
	21	22
8	19	21
	21	22
	21	23
	22	23
9	20	22
	20	22
	20	23
	19	21
12	20	24
	20	19
	21	15
	21	22

Fig. 3B

	Blood glucose (mg/dl)					
**DIET**	**0'**	**15'**	**30'**	**60'**	**90'**	**120'**
HFD	60	106	85	58	50	35
D12492	66	140	134	87	82	68
	94	177	141	125	94	87
	92	202	155	109	83	73
CD	64	172	151	100	84	76
D12450K	79	112	112	99	64	68
	24	121	154	116	69	25
	96	197	180	148	110	106

Fig. 3C

	Fed glucose (mg/dl)
HFD	113
D12492	95
	101
	114
CD	107
D12450K	116
	158
	127
